# Primary Synovial Sarcoma of the Kidney: Diagnostic Approach and Therapeutic Modalities for a Rare Nosological Entity

**DOI:** 10.3390/jpm12091450

**Published:** 2022-09-02

**Authors:** Aikaterini Mastoraki, Dimitrios Schizas, Despoina Maria Karavolia, Antonios Smailis, Nikolaos Machairas, Michail Vailas, Adamantios Michalinos, Dimitrios Tsapralis, Ioannis Anastasiou, George Agrogiannis

**Affiliations:** 1 First Department of Surgery, National and Kapodistrian University of Athens, Laikon General Hospital, 11527 Athens, Greece; 2 Second Department of Propaedeutic Surgery, National and Kapodistrian University of Athens, Laikon General Hospital, 11527 Athens, Greece; 3 Department of Anatomy, European University of Cyprus, 1516 Nicosia, Cyprus; 4 Department of Surgery, General Hospital of Ierapetra, 72200 Ierapetra, Greece; 5 First University Urology Clinic, National and Kapodistrian University of Athens, Laikon General Hospital, 11527 Athens, Greece; 6 First Department of Pathology, Medical School, National and Kapodistrian University of Athens, 11527 Athens, Greece

**Keywords:** retroperitoneal, synovial, sarcoma, therapeutic management, prognostic parameters

## Abstract

Synovial sarcoma (SS) is a rare mesenchymal entity that represents 5–10% among soft tissue sarcomas (STS). Primary renal synovial sarcoma (PRSS) is an uncommon, rapidly growing tumor, with potential metastatic dissemination. The main prognostic factors of PRSS include tumor size and histologic grade, while translocation t (X; 18) (p11.2; q11.2) (fusion of SYT gene -chromosome 18- with SSX genes (1, 2 or 4)-chromosome X) is the most common pathognomonic sign. Aggressive surgical resection of the tumor along with concomitant regional lymphadenectomy is the treatment of choice for PRSS, while additional en bloc resection of the adjacent affected organs is often performed. To date, the role of preoperative or postoperative chemotherapy remains equivocal. The prognosis of patients with PRSS is poor, as the 5-year survival rate is only 20–30% and further deteriorates when a high mitotic activity is detected. Local recurrence even after complete R0 surgical excision remains the most frequent cause of death. The aim of this review was to meticulously discuss clinical features, histogenesis, and morphological and immunochemical findings of PRSS, while the role of current diagnostic and therapeutic management of this aggressive neoplasm was emphasized.

## 1. Introduction

Synovial sarcoma (SS) is a rare mesenchymal entity that represents 5–10% among soft tissue sarcomas (STS) [1]. Lejars and Rubens-Duval reported and initially named it synovial endothelioma in 1919. The most predominant theory for its origin is the retrograde differentiation of an undefined mesenchymal cell [2,3]. This type of tumor can be encountered either in the extremities close to articulations (85–95%) or bursas, sinews and the head and neck region (10%) [4]. It also can be detected in unusual parts of the body without correlation to the joints, including the nervous system, thoracic and abdominal wall cavity, prostate, fallopian tubes, retroperitoneum, bones and kidneys [4,5,6,7,8].

Primary renal synovial sarcoma (PRSS) presents an incidence of 1–3% among all renal tumors [9]. The first description was in 1999 by Faria, while it was previously categorized as an embryonal sarcoma of the kidney [10,11]. Clinical features of PRSS typically range from presence of an enlarged abdominal mass, vague pain and hematuria to local invasion as well as liver and lung metastatic disease [12,13,14,15,16]. Due to the limited number of sporadic cases of PRSS, standard protocols for the diagnosis and treatment of this rare neoplasm are strongly required [17,18,19,20]. The aim of this review was to meticulously discuss clinical features, histogenesis, and morphological and immunochemical findings of PRSS, while the role of current diagnostic and therapeutic management of this aggressive neoplasm was emphasized.

## 2. Epidemiology and Classification

STS is a rare mesenchymal malignancy that accounts for less than 1% of all adult tumors and affects 2–3 per 100,000 people. In addition, 5–10% of all STSs are represented by SS, which can rarely be encountered in the kidney [20,21]. PRSS is the fourth most common subtype of primary sarcoma of the kidney following undifferentiated pleomorphic sarcoma, liposarcoma and rhabdomyosarcoma [9]. To date, only 185 cases have been reported in the literature, since the first description of PRSS in 1999 [20,22,23,24,25]. Notably, the translocation t(X; 18) (p11.2; q11.2) is present in around 95% of the affected patients [26,27].

The above-mentioned cases were recently systematically reviewed, and more comprehensive epidemiological data were revealed [25]. In that study the median age of PRSS patients was 36.2 years, and the male to female ratio was 1.14:1. The main localization of tumor was in the right kidney with a right to left ratio of 1.53:1. At the time of diagnosis, 65% of affected patients were symptomatic, 46.3% appeared with hematuria, and 43% with pain. Moreover, 8% of the patients presented with metastatic disease, whereas there was caval thrombus formation in 48.2% of the cases [12,25,28,29]. As reported, the lungs are the most common sites for distant metastasis, while the median survival time is 34 months [25,29].

PRSS develops in three possible histological patterns: the monophasic (MSS), the biphasic (BSS), and the poorly differentiated type (PDSS) [8,17]. Relevant prevalence of the above-mentioned PRSS classifications has been identified in 76%, 14% and 10% of cases, respectively [23,25]. Firstly, MSS reveals, microscopically, ovoid or spindled cells with moderate nuclear pleomorphism, arranged in solid compact collagenous sheets without epithelial cell component, and abundant solitary fibrous tumor-like vessels in the background. The monophasic type may be misinterpreted with spindle cell tumors, such as fibrosarcoma, malignant peripheral nerve sheath tumors, adult Wilms’ tumor and vessel-derived tumors such as solitary fibrous tumor. BSS combines MSS’ microscopic features and spindle cells with glandular elements arrayed with mucosal epithelium [14,20,22,27]. It is the pattern most easily distinguishable from other tumors and expresses the SYT-SSX1 fusion, while the monophasic form shows the SYT-SSX2 fusion. Other than that, both MSS and BSS have the same clinical, ultrastructural, and molecular features [30,31]. On the other hand, PDSS includes undifferentiated round cells and is characterized by increased mitotic rate with the poorest prognosis [12,22].

Three PDSS variants have been described, including a subtype of large cells (with fusiform, epithelioid and rhabdoid characteristics), a type of small cells (comparable to Ewing sarcoma and NETs) and a fusocellular category (similar to fibrosarcoma and malignant peripheral nerve tumors). However, PDSS can be more easily distinguished because of the presence of cellular areas with hypocellular zones with calcification or hyalinization and mast cells near fusiform cells [3]. Lastly, there is a TNM classification by the American Joint Committee on Cancer for all the primary renal sarcomas, but its use for staging sarcomas does not sufficiently predict patient prognosis. Furthermore, the histological grade can be adequately determined based upon the scoring system of the French Federation, although SS is considered as grade 3 by definition [32].

## 3. Clinical Features

The clinical features of PRSS are not specific, making its differential diagnosis from other renal tumors challenging at an early stage. The most commonly reported symptom is gross, macroscopic hematuria, which sometimes occurs painlessly [25,33,34,35,36]. Another clinical sign is intermittent mild to intense abdominal or lumbar pain [37]. In asymptomatic patients, a bimanually palpable non-tender abdominal mass, with smooth surface and firm to hard consistency, can be detected incidentally during routine evaluation. The lump may be located at the right or left hypochondrium as well as the lumbar, extending into the iliac region [1,30,31]. Other, less frequent symptoms include discomfort, fullness, unwilling weight loss, low-grade fever, hypertension, discolored urine, dysuria, pallor in the sclera, signs of inflammation, nausea and vomiting [19,21,26,35,38]. A rare case of jaundice in a patient with Stauffer’s syndrome has been reported in the literature as a paraneoplastic manifestation of PRSS [37].

The most frequent sites of metastasis are the lungs (36.4% of the patients regardless of the fusion type), with hemoptysis and breathlessness as the main symptoms. Other metastatic foci occur at the liver (50% of patients with SYT-SSX2 fusion type), bones, perirenal adipose tissue and brain [20,22,25,29,39,40]. The tumor metastasizes via the blood and rarely via the lymphatic system. It mainly appears at renal hilar, aortocaval and aorto-iliac lymph nodes [20,22,25]. Moreover, approximately 15% of patients are diagnosed with formed thrombus in adjacent vessels including the renal vein, inferior vena cava (IVC) and portal vein or have a thrombus in the right atrium. Symptoms including non-pitting pedal edema over the shin, non-reducing varicocele and pulmonary embolism are the result of vascular metastasis [14,22,25,35,41]. Nevertheless, despite achievement of adequate resection margins, PRSS is shown to recur in adjacent and distant parts of the body at a percentage of 30–50% [30]. Disease-free survivals range from 5 to 32 months [22].

## 4. Histopathology and Immunochemistry

Macroscopically, PRSS presents as a yellow-brown, jelly-like, well-encapsulated mass [1,15,42] consisting of cystic, necrotic and hemorrhagic areas [30]. Tumor size ranges variably from 1–35 cm, and most commonly varies from 5–20 cm [12,25,31,43]. It can replace the whole kidney, as well as penetrate the nearby focal renal capsule [10]. Although enlargement of lymph nodes is rare, vascular invasion can be encountered [10,23]. The most common microscopic components are the spindle cells arranged in solid, short, intersecting fascicles with oval to spindle hyperchromatic mitotically active, pleomorphic nuclei and scant to moderate eosinophilic cytoplasm. The nucleus to cytoplasm rate is high. Frequently, the cells develop a perivascular pattern. Thick wall cystic areas lined by hobnailed epithelium are also typical. The tumor is characterized by dilated renal tubules, necrosis, hemorrhage, minimal fat and extensive neovascularization [22,23,24,27,30,35,44]. In the BSS type, secondarily, a degree of epithelial differentiation with microcyst formation coexists, while in PDSS, immature cells with irregular nuclei replace the renal parenchyma [3,36].

**Figure 1:**
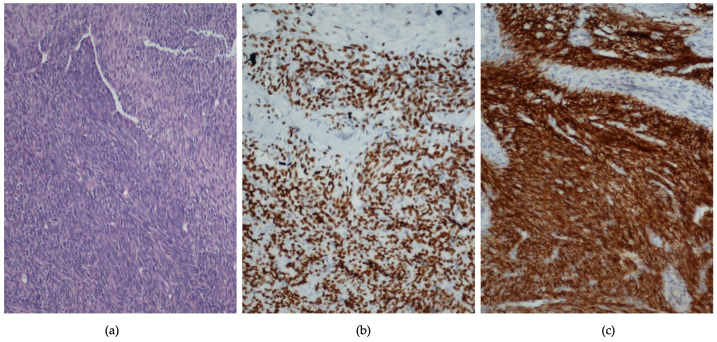
A monophasic PRSS. (**a**) Hematoxylin & eosin stain (x200). (**b**) TLE-1 stain (x200). (**c**) CD56 stain (x200).

In addition, there is an optimal immunohistochemical (IHC) panel of markers that can be used to establish SS diagnosis (Figure 1). The markers, which are usually positive in an IHC test, are epithelial membrane antigen (EMA), cytokeratin (CK) 7, CK/MNF116, B cell lymphoma 2 (BCL2- associated with the SYT-SS2 fusion and may indicate ineffectiveness of chemoradiation after surgery), cluster of differentiation molecule 99 (CD99/MIC2), CD56, Ki67, vimentin and transducin-like enhancer of split 1 (TLE1). Mostly negative markers are CD34, Desmin, Wilms’ tumor protein (WT-1), smooth muscle antibody (SMA), MyoD1, S100, paired-box factor 8 (PAX8—regularly positive in renal cell carcinoma) and synaptophysin [17,31,43,44,45,46,47,48].

Especially, TLE1 has the highest negative predictive value, specificity and sensitivity among all currently available IHC test markers (Figure 1). Nevertheless, it can be detected in many other mesenchymal tumors. This means that a TLE1 negative result excludes PRSS from the differential diagnosis [20,49]. However, FISH still remains the gold standard technique for spotting the pathognomonic SYT-SSX fusion oncogene [1,50,51].

## 5. Diagnostic Modalities

Several imaging techniques have been proposed to more reliably diagnose PRSS [10,15]. In ultrasound (U/S) images, SS is usually depicted as a single or multiple solid hypo-echoic masses, with unclear or irregular boundary, with additional calcifications, lymph node infiltration or pseudocapsule. Alternative U/S variants are highly recommended; color Doppler flow imaging (CDFI) is indicative of hypovascularity, dotted blood flow signal inside the neoplasm, and on occasion, renal vein thrombosis [14]. Intraoperative transesophageal echocardiography (TEE) can detect extensions such as cardiac metastasis. A common early finding is also bleeding of the ureter in U/S cystoscopy. Lastly, although contrast enhanced ultrasound (CEUS) reveals “slow in and fast out” enhancement, which is unusual in other renal tumors, parametric imaging with software such as “SonoLiver CAP” is proposed to conduct further quantitative analysis of the SS’s enhancement pattern [16,23,52].

**Figure 2:**
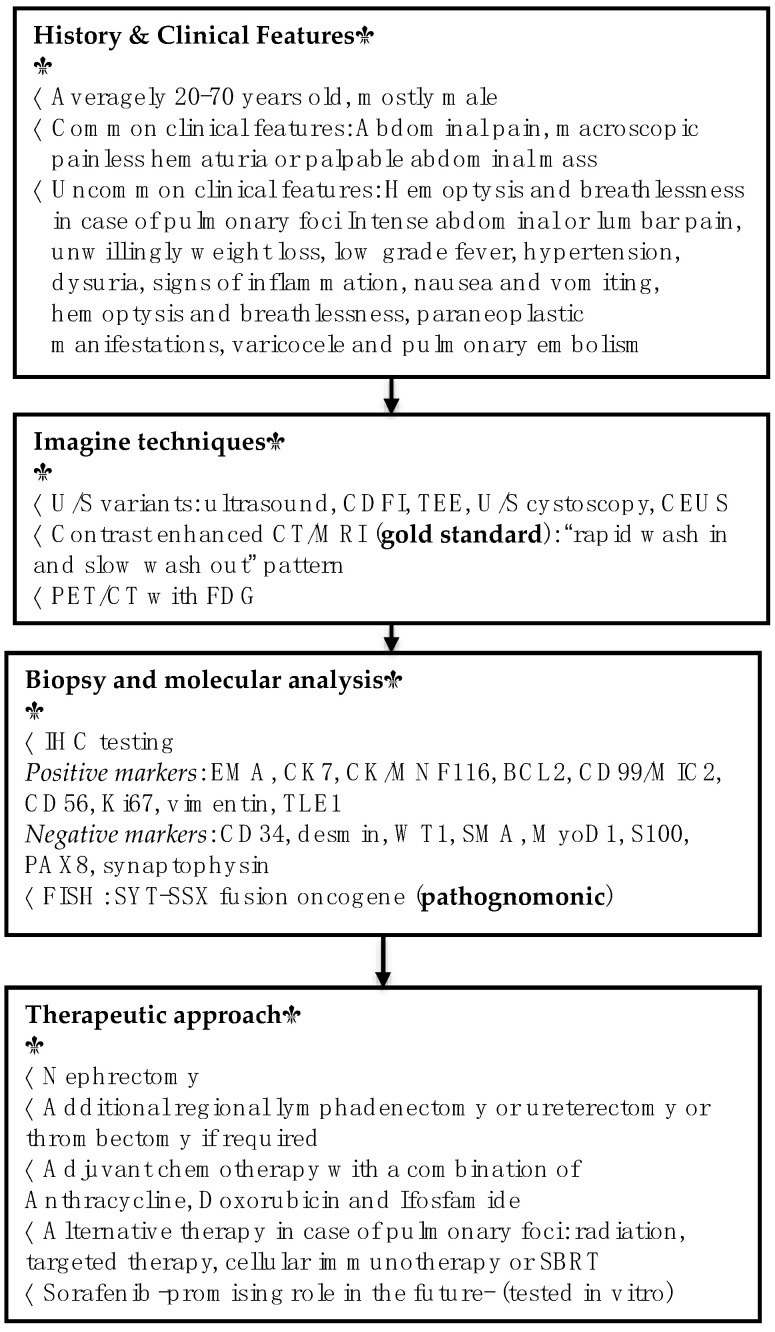
Diagnostic and therapeutic algorithm for PRSS.

Computed tomography (CT) and magnetic resonance imaging (MRI) scans usually reveal an enhancing renal mass with solid and cystic components and unclear margins that may extend to the perinephric area [53]. Additional intravenous contrast administration helps distinguish solid components with heterogeneous enhancement that lasts during nephrography and excretory phase and follows the “rapid wash in and slow wash out” pattern. However, MRI is the gold standard imaging modality for soft-tissue tumors and, therefore, is preferred for sarcomas [54]. On T1-weighted MRI sequences, the tumor is hypointense, similarly to paraspinal muscles. T2-weighted MRI depicts hyperintense areas with the “triple sign” (hemorrhage/necrosis, calcification/fibrosis and air-fluid levels), which can raise suspicions for a preoperative diagnosis. Both CT and MRI can provide critical information on the disease extent, including presence of lymphadenopathy, formation of pseudocapsule or subcapsular hematomas, thrombus in renal veins and local infiltration. The role of nuclear imaging for detecting PRSSs remains ill-determined. A few studies assessed the superiority of positron emission tomography (PET)/CT for the detection of local recurrence and metastasis and, in particular, they indicate multiple hyper-density entities with increased fluorodeoxyglucose (FDG) metabolism in the primary tumor and metastatic lymph nodes, especially in the delayed phase [55]. Diagnostic as well as therapeutic algorithms are summarized in Figure 2.

Tissue biopsy and molecular analysis suggest useful diagnostic approaches [56,57]. Fine needle aspiration cytology (FNAC) or biopsy (FNB) is useful for preoperative diagnosis. Tissue biopsies, though, with IHC markers, are safer to confirm suspicion of PRSS, especially in cases of retroperitoneal masses without typical imaging characteristics. It is indisputable that marking the SYT-SSX oncogene with FISH is a pathognomonic tool for establishing SS diagnosis [58,59]. However, it is reported that some cases of SS do not express the characteristic translocation, and the combination of histopathological features with the clinical picture and imaging is warranted. In one case without the SYT-SSX gene and in some cases with certain SYT-SS2 genes, the prognosis for inducing remission was better after chemotherapy [60].

Differential diagnosis includes primary renal tumors including fibrosarcoma, sarcomatoid renal cell carcinoma, solitary fibrous tumor, adult Wilms’ tumor, primary renal primitive NETs, undifferentiated carcinoma, congenital mesoblastic nephroma, sarcomatoid transitional cell carcinoma of the renal pelvis, and angiomyolipoma [15,16]. Secondary tumors that should also be considered are primary retroperitoneal sarcoma involving the kidney, renal metastatic or Ewing sarcoma, and NETs. By definition, fibrosarcomas are only immunohistochemically reactive for vimentin and rarely for SMA. Biphasic and monophasic SS can be closely similar to sarcomatoid renal cell carcinoma, which has a honeycomb pattern of enhanced signal in CEUS. On the other hand, SS mitotic activity is elevated compared to solitary fibrous tumor, which is positive for CD34 and pSTAT6 and negative for CK [61]. The ETV6-NTRK3 fusion gene can be detected by polymerase chain reaction (PCR) to confirm the diagnosis of congenital mesoblastic nephroma [62,63].

## 6. Therapeutic Approach

Due to the rarity of PRSS and the limited cases reported in the literature, there are no specific guidelines for its therapeutic management. All cases of suspected sarcoma need to be reviewed by a multidisciplinary sarcoma team/center before initiating treatment (Figure 2).

The main approach consists of surgical treatment followed by adjuvant chemotherapy. The vast majority of centers would consider pre-operative chemotherapy and radiotherapy for SS of the retroperitoneum such as PRSS, if it is likely to reduce the morbidity of a radical surgical resection (organ preserving or aiding marginality against a vital structure). Patients undergo nephrectomy to alleviate the symptoms of PRSS and prevent local relapse with radical resection [29,37,64]. A thoracoabdominal incision with partial resection of the diaphragm and ascending colon is sometimes indicated for radical surgical intervention. Emergency laparotomy may also be performed in case of severe abdominal pain or hemorrhage. Nevertheless, in cases of major vessel invasion or gross hematuria with active bleeding, an embolization may be performed preoperatively. Finally, laparoscopic surgery has been suggested as an alternative treatment option for selected cases.

More than 90% of patients with completely resected PRSSs will relapse locally. Unfortunately, from published series, it is unclear which tumors will recur after total resection even with negative microscopic margins. The optimal extent of surgical margins required to achieve tumor clearance remains largely unknown. Additionally, the role of partial resection in these highly vascular and frequently necrotic tumors remains to be determined [1,13,65]. Intra-operatively, the adrenal gland is preserved when feasible [37,38]. Additional regional lymphadenectomy or ureterectomy is performed as required [6,45,55]. As previously mentioned, vascular thrombi are common and may extend into the infra-hepatic IVC and left lumbar vein [14,23,29,30,52]. In the latter case, they must be excised through thrombectomy.

The need to establish certain diagnostic and therapeutic guidelines (including examination of imaging, radiomics, tumor biology, and surgical and chemotherapeutic tools for retroperitoneal sarcomas, such as PRSS) led to the establishment of the retroperitoneal sarcoma registry “RESAR” between centers in Europe, North America, Asia, Australia and South America. New prospectively collected registry data are expected to be used in auxiliary studies [66].

The clinical benefit of adjuvant chemotherapy remains controversial and is studied with new registry data. Initial studies included anthracycline- and ifosfamide-based chemotherapy and revealed a small benefit in terms of survival, which, however, was not reproduced in a subsequent, large clinical trial [59,66]. Thus, no consensus has been yet achieved, and the debate is still ongoing, whereas the chemotherapeutic management of PRSS varies between institutions and countries. So far, there is no convincing evidence on survival benefits of chemotherapy (for the different histological subtypes of SS), the quality of treatment, and the criteria for selection of patients for adjuvant systemic therapy [26]. Therefore, recent surveys recommend the use of adjuvant chemotherapy only for younger patients and/or larger tumors where clinical advantages could rather be expected. In general, RSS is sensitive to chemotherapy in up to 53% of cases, leading to an overall survival benefit of 8–10% [56]. The use of chemotherapy preoperatively reduces the size of the mass up to 50%, facilitating the following surgical manipulations. Postoperative chemotherapy increased the time of relapse, as well as general survival [14,16]. The most common chemotherapeutic regimen consists of a combination of anthracycline, doxorubicin and ifosfamide in a total of 3–6 cycles [20,21]. Recent investigations indicated that doxorubicin-based chemotherapy significantly increased time of local and distant recurrence, as well as overall recurrence-free survival in comparison to patients who were just observed without therapy. However, an increase in overall survival was not statistically significant [58].

Adjuvant radiotherapy may be used to help local control of high-grade, superficial lesions of the tumor. Radiation can be beneficial in decreasing local recurrence rates and metastases but may not be a feasible option if there is a risk of radiation injury to an adjacent organ. The benefit of radiotherapy in synovial sarcoma is less clear than for chemotherapy. Nevertheless, in case of pulmonary metastatic foci, stereotactic body radiotherapy (SBRT) constitutes an additional therapeutic approach with comparable results and prognosis to surgical resection. In the SBRT regimen, three-dimensional image-guided high-dose radiation is administered within a course of treatment of up to five fractions [9,10,15,40].

**Figure 3:**
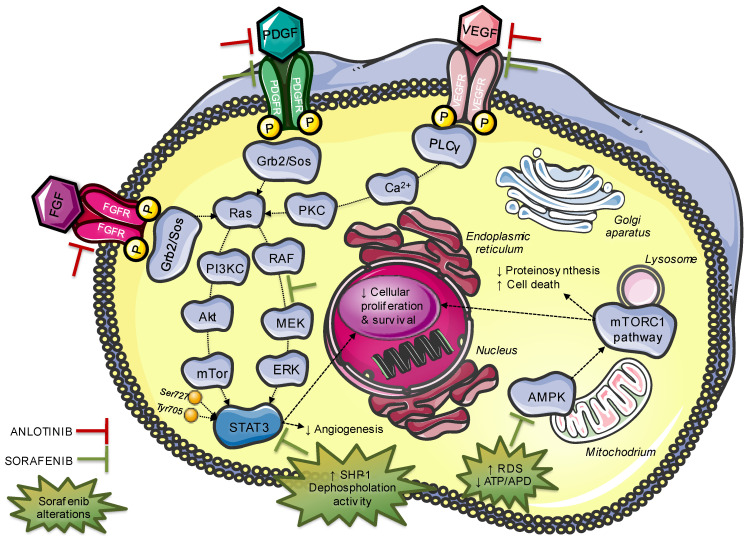
Genetic pathways and regulatory mechanisms modified by the administration of immunotherapeutic agents.

Alternative therapeutic options include Anlotinib as a targeted therapy with adequate response in decreasing the lung metastatic nodules. Anlotinib is a novel tyrosine kinase inhibitor targeting multiple factors involved in tumor proliferation, vasculature and tumor microenvironment (Figure 3). Anlotinib inhibits VEGF/VEGFR signaling by selectively targeting VEGFR-2, -3 and FGFR-1, -2, -3,-4 with high affinity, leading to significant inhibition of tumor proliferation (Figure 2). Therefore, in patients with several metastatic SS entities who were refractory to previous anthracycline-based chemotherapy, Anlotinib was proved to have broad-spectrum antitumor activity, while its toxicity was manageable and acceptable [67]. Similarly to treatment for other types of tumors, immunotherapy is also used for patients with STS [68,69]. In this case, autologous T-cells, transduced with a T-cell receptor on tumor-infiltrating lymphocytes, target the NY-ESO-1 antigen. The latter has been detected in 80% of SS affected patients and revealed as an excellent target for NY-ESO-1 positive sarcomas [51].

Sorafenib is another potential future therapeutic choice that could be per-os administered (Figure 3). Mitogen-activated protein kinase (MAPK) cascades have been shown to play a pivotal role in SS survival. Sorafenib, a potent recombinant activated factor (RAF) inhibitor, effectively inhibits the MAPK signaling pathway and cellular proliferation and induces apoptosis, downregulating cyclin D1 and Rb levels (Figure 3). In addition, the above-mentioned compound inhibits the tyrosine kinase receptors VEGF and PDGFR and blocks the serine/threonine kinase RAF, a mediator of IGF-induced signal transduction. The transcription of IGF-2 is activated by SS18-SSX fusion proteins and may be encountered in aggressive types of SS. Therefore, the abeyance of the RAF/MEK/ERK signaling pathway inhibits the proliferation of SS cell lines [15,70,71]. Finally, sorafenib downregulates cyclin D1, whose accumulation is also promoted by SS18-SSX fusion proteins, resulting in G1 arrest and S phase decrease (Figure 2). In conclusion, sorafenib seems to be effective for growth inhibition of SS cell lines in vitro and may become a new therapeutic option for patients with synovial sarcoma [72]. However, more clinicals trials are warranted in order to estimate the efficacy of sorafenib.

## 7. Conclusions

PRSS is a rare, rapidly growing tumor, with potential metastatic dissemination. Tumor size and histologic grade are the main prognostic factors of PRSS, while aggressive surgical resection of the tumor remains the treatment of choice. The role of preoperative or postoperative chemotherapy remains uncertain. Neoadjuvant chemotherapy may reduce the size of the tumor and simplify subsequent surgical operations, especially in large and high-grade tumors. In addition, patients who receive adjuvant chemotherapy exhibit improved disease-free survival rates. Radiation therapy alone as a complementary treatment modality to surgery either before or after resection results in non-significant differences in survival rates, whereas neoadjuvant administration of chemotherapy combined with radiation may be beneficial. So far, there is no convincing evidence on survival benefits of chemotherapy (for the different histological subtypes of SS), the quality of treatment and the criteria for selection of patients for adjuvant systemic therapy. Finally, emerging data support resection of pulmonary and hepatic metastases, which may improve survival in selected cases. The prognosis of patients with PRSS remains dismal, as the 5-year survival rate is only 20–30% and deteriorates when high mitotic activity is presented. Local recurrence even after complete R0 surgical excision remains the most common cause of death. Evaluation of patients from experienced centers in the context of MDT decision-making is of cardinal importance in order to provide the optimal treatment planning and subsequently the best possible long-term outcomes.

## Data Availability

Not applicable.
